# Performance Analysis of Loose-Fill Thermal Insulation from Wood Scobs Coated with Liquid Glass, Tung Oil, and Expandable Graphite Mixture

**DOI:** 10.3390/ma16093326

**Published:** 2023-04-24

**Authors:** Nerijus Augaitis, Jurga Šeputytė-Jucikė, Sylwia Członka, Arūnas Kremensas, Agnė Kairytė, Sigitas Vėjelis, Giedrius Balčiūnas, Saulius Vaitkus

**Affiliations:** 1 Laboratory of Thermal Insulating Materials and Acoustics, Institute of Building Materials, Faculty of Civil Engineering, Vilnius Gediminas Technical University, Linkmenų st. 28, LT-08217 Vilnius, Lithuania; jurga.seputyte-jucike@vilniustech.lt (J.Š.-J.); arunas.kremensas@vilniustech.lt (A.K.); agne.kairyte@vilniustech.lt (A.K.); sigitas.vejelis@vilniustech.lt (S.V.); giedrius.balciunas@vilniustech.lt (G.B.); saulius.vaitkus@vilniustech.lt (S.V.); 2 Institute of Polymer and Dye Technology, Lodz University of Technology, Żeromskiego 116, 90-924 Lodz, Poland; sylwia.czlonka@edu.p.lodz.pl

**Keywords:** biocomposites, loose-fill thermal insulation, thermal conductivity, three-component coating, wood scobs, water absorption

## Abstract

The current study presents the results of monitoring the behavior of loose-fill thermal insulating material for buildings made of wood scobs (WS), which were coated with one, two, and three component-based coatings from liquid glass (LG), tung oil (TO), and expandable graphite (EG). The thermal conductivity of samples in the dry state and under normal laboratory conditions, short-term water absorption by partial immersion, surface wettability, and water vapor permeability were evaluated, and regression equations describing the variations in numerical values of specified properties under different amounts of each coating component were presented. It was shown that LG and TO act as hydrophobic layers that, in conjunction, reduce water absorption by a maximum of 274%, have a contact angle equal to 86°, and lower thermal conductivity by 55% in the dry state due to the specifics of the layer formed on the surface of WS. The addition of EG to LG coating resulted in insignificantly changed water absorption and thermal conductivity values, indicating the potential of this material to be used to improve the fire resistance of wood-based composites in the future. The results showed that the three-component layer of LG/TO/EG reduces water absorption by a maximum of 72%, increases thermal conductivity in the dry state by a minimum of 0.4%, and increases the contact angle to 81° at 100 wt.% LG. The changes in water vapor permeability of all compositions were determined to be insignificant.

## 1. Introduction

It is confirmed that most existing European buildings could be more energy efficient [1]. That is why the European Commission proposed the Climate Target Plan 2030 to reduce greenhouse gas emissions by 55% in comparison to 1990 [2]. Many use fossil fuels for cooling and heating and are still using polluting appliances and outdated technologies. Therefore, energy deprivation is still a crucial challenge for many Europeans. Generally, buildings account for approximately 40% of Europe’s total energy consumption, of which 36% are energy-related greenhouse gas emissions [3]. Europe must make all efforts to make buildings more sustainable, less carbon intensive, and more energy efficient. The possible solution is to apply circular economy principles to building strategies and renovation, thus reducing building materials related to greenhouse gas emissions. Regarding the ongoing greenhouse gas reduction plan, the Ministry of Environment of the Republic of Lithuania formed a work group that suggested renovating old buildings using building materials consisting of not less than 50% wood-based and organic materials [4]. Although some definitions of what can be applied must be clarified, the building sector in Lithuania is progressing for the upcoming changes in 2024.

Traditional building products are well known, tested, and certified, and their quality control is performed periodically, while the introduction of new building materials such as wood or wood waste-based composites in the market is complicated as they do not have harmonized product standards. Additionally, intrinsic properties make wood, or wood waste such as scobs, sawdust, or chips, one of the natural materials that are increasingly being researched by industry and academia [5,6,7]. Because of its sufficient mechanical performance, lightness, thermal insulating properties, ease of processing, relatively low cost, and eco-friendly nature, wood has been implemented as a raw material in the building industry. However, the use of wood in some application areas can be challenging due to its susceptibility to moisture/water, UV, fungi, and flammability. In order to overcome some of the mentioned drawbacks, a few successful approaches have been suggested, such as superhydrophobic coatings from plant oils and natural wax [8], zinc oxide and cerium oxide nanoparticles embedded in linseed oil nano-emulsion coatings for UV resistance [9], coatings with soybean protein isolate nano-silver hydrosol with antibacterial properties [10], and vanillin-based epoxy coatings for fire resistance [11]. Although these novel approaches highly contribute to the science of wood coatings, they have been mostly developed to have a specific significance in improving targeted performance characteristics. Currently, the synergy effect between two or more raw materials bringing out their best properties is increasingly used to improve all kinds of products. Therefore, the proposed version of wood coating in the current study is a system consisting of liquid glass as a wood preservative against fungal attack and as a fire retardant [12], tung oil as a component system that promotes hydrophobicity and reduces hygroscopicity [13], and expandable graphite as an antibacterial and antifungal agent with an intumescent flame retardant [14,15]. 

The aim of the current study is to propose an alternative to traditional eco-wool loose-fill thermal insulating material made from pine wood scobs (WS) coated with a system consisting of liquid glass (LG), tung oil (TO), and expandable graphite (EG). Different amounts of each raw material were involved to determine the optimal composition. The efficacy of each coating and filler was evaluated concerning structural changes, the thermal conductivity of dried composite material, and the material under normal laboratory conditions. Furthermore, short-term water absorption by partial immersion, surface wettability via water contact angle measurements, and water vapor permeability were also evaluated. Additionally, statistical-mathematical processing of the results and the resulting regressions were presented. Although expandable graphite was incorporated as an intumescent flame retardant, thermal stability, fire resistance, and ignitability studies were not implemented and are currently under evaluation for future research work.

## 2. Materials and Methods

### 2.1. Materials

Sodium silicate solution-liquid glass (LG) was purchased from JSC Lerochem, Klaipėda, Lithuania. LG contains 9.3% Na_2_O and 28.0% SiO_2_, has a molar ratio of 3.1, and has a density at 20 °C equal to 1.38 g/cm^3^. TO was supplied by JSC Pro Colore, Vilkaviškis, Lithuania. EG ES 350 F5 was purchased from Qingdao Kropfmuehl Graphite Co., Ltd., Qingdao, China, with the following characteristics: 98% carbon content, 350–700 cm^3^/g expansion rate, and a starting temperature of 180–240 °C. Pine WS were received from the Dzukija region forest in Alytus, Lithuania, with a bulk density of 83.0 ± 6.8 kg/m^3^, moisture content of 12.1 ± 1.9%, and the following granulometry: 3.64% (≥10 mm), 43.4% (10–5 mm), 30.4% (5–2.5 mm), 14.3% (2.5–1.25 mm), 6.56% (1.25–0.63 mm), 0.81% (0.63–0.315 mm), 0.89% (<0.315 mm).

### 2.2. Preparation of Loose-Fill WS Composites

LG solution, LG/TO, LG/EG, and LG/TO/EG suspensions were prepared according to the compositions indicated in Table 1. 

The respective amounts of each component were weighted and mixed with a high-speed mixer for 1 min at 16,000 rpm. Before the application of LG solution or LG/TO, LG/EG, or LG/TO/EG suspensions, WS particles were conditioned for 72 h at 23 ± 5 °C and 50 ± 5% relative air humidity. Further, the prepared LG solution and suspensions were sprayed on the scaled amount of WS and thoroughly hand-mixed. Then, the prepared coated WS samples were vacuumed for 30 min at 1 bar and dried at normal laboratory conditions, i.e., 23 ± 5 °C temperature and 50 ± 5% relative air humidity conditions, for 7 days.

### 2.3. Methods

The microstructure studies of the WS particles and LG/TO/EG mixture-coated WS particles were conducted using a scanning electron microscope (SEM) Helios NanoLab 650 (Oxford Instruments, Abingdon, UK), which has a magnification of up to 1 million times. Before the test, the prepared WS, WS-LG/TO/EG samples were coated with a thin gold layer using vacuum with a Quorum Q150R ES instrument (Quorum, Laughton, UK). Two samples for each coating solution were tested.

The chemical structure of control WS and selected WS composites was evaluated with Fourier Transform Infrared Spectroscopy (FTIR). The apparatus implemented for the study was a Nicolet iS50 spectrometer (Thermo Fisher Scientific, Waltham, MA, USA).

Thermal conductivity measurements of dried samples (at 70 °C temperature) and samples under normal laboratory conditions (23 ± 5 °C temperature and 50 ± 5% relative air humidity) were conducted at an average testing temperature of 10 °C on 300 × 300 × 50 mm^3^-sized samples based on EN 12667 [16] requirements using a heat flow meter FOX 304 (TA Instruments, New Castle, DE, USA) with active edge insulation. The direction of the heat flow was upwards during the test, and the difference between the cold and hot plates was 20 °C. Three samples for each coating solution were tested.

The short-term water absorption of the control loose-fill WS and LG/TO/EG mixture coated WS composites was carried out according to EN ISO 29767 [17] for samples with a mesh size of 200 × 200 × 50 mm^3^. The test was carried out for 24 h by partially (10 ± 1 mm) immersing samples into the 20 °C water. After the test, samples were drained of excess water for 10 min using a drainage stand. Five samples for each coating solution were tested.

A water vapor permeability test was carried out for samples having a mesh and a loose-fill sample size of 100 × 100 × 50 mm^3^ based on EN 12086 [18] requirements. Mesh assemblies with samples and salt were maintained at 23 ± 1 °C temperature and 50 ± 3% moisture. The required moisture of 0% in the assemblies was achieved with calcium chloride. Five samples for each coating solution were tested.

The surface hydrophobicity was determined by contact angle measurements using the sessile drop method. The examination was performed using a manual contact angle goniometer with an OS-45D optical system (Oscar, Taiwan) to capture the shape of liquid on the solid surface. Water drops of 1 µL were deposited using a micrometer syringe fitted with a stainless-steel needle onto the flat surfaces of control WS and WS-LG/TO/EG composites. The results were taken instantly after water droplet deposition. The contact angles were measured at least five times on each sample and averaged. Two samples of the selected coating solution were tested.

## 3. Results and Discussion

### 3.1. Thermal Conductivity and Structural Changes of Control WS and Modified WS Composites

**Figure 1:**
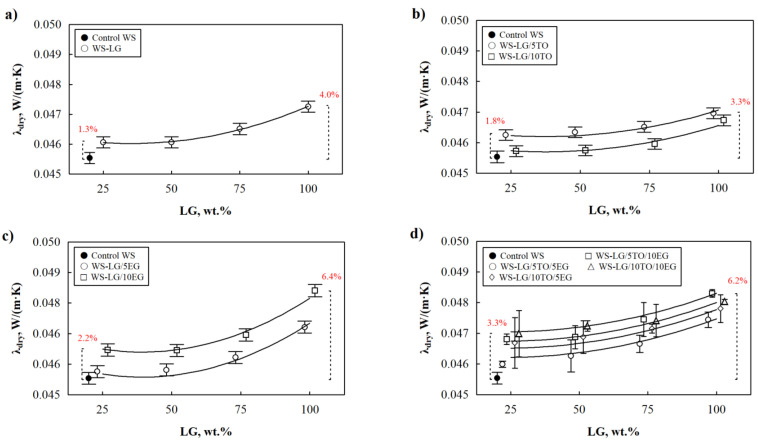
Thermal conductivity of WS-LG, WS-LG/TO, WS-LG/EG, and WS-LG/TO/EG composites after drying at 70 °C: (**a**) WS-LG composites; (**b**) WS-LG/TO composites; (**c**) WS-LG/EG composites and (**d**) WS-LG/TO/EG composites.

For real-world applications, thermal characteristics are another essential measurement of thermal insulating materials. Therefore, Figure 1 shows the impact of LG coating on the thermal conductivity of dried at 70 °C WS-LG composites. It can be clearly seen from Figure 1a that the lowest thermal conductivity value is obtained for control WS, while the lowest value for WS-LG composites is at 25 wt.% LG coating, which is 1.3% higher compared to control WS. Moreover, further addition of up to 100 wt.% LG gradually increases the parameter to 4% compared to control WS.

**Figure 2:**
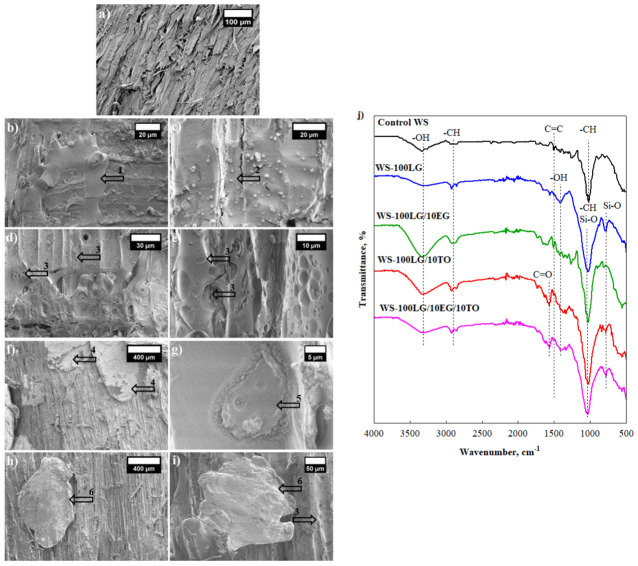
Microstructural changes of control WS and modified WS composites: (**a**) control WS (magnification ×200); (**b**) WS-50LG (magnification ×1500); (**c**) WS-100LG (magnification ×2000); (**d**) WS-50LG/10TO (magnification ×1500); (**e**) WS-100LG/10TO (magnification ×1500); (**f**) WS-50LG/5EG (magnification ×100); (**g**) WS-100LG/5EG (magnification ×5000); (**h**) WS-50LG/10TO/5EG (magnification ×100); (**i**) WS-100LG/10TO/5EG (magnification ×500); (**j**) FTIR spectra (1—uneven LG layer; 2—even LG layer; 3—TO drop in LG layer; 4—EG on WS surface; 5—EG in LG layer; 6—EG in LG and TO layer).

The change in thermal conductivity of WS-LG composites is not a surprise and can be explained by the fact that LG has a higher thermal conductivity value. Therefore, incorporating raw materials with higher heat conduction increases the thermal conductivity value of the whole system [19,20]. In order to evaluate the synergistic effect between two coating materials on the thermal conductivity of dried composites, 5 wt.% and 10 wt.% TO are incorporated in addition to LG. Figure 1b indicates that 10 wt.% is an efficient amount for TO to reduce the thermal conductivity of dried WS-LG composites. Together with LG, 10 wt.% TO reduces the parameter of WS-LG/10TO composites by a maximum of 1.2% compared to WS-LG composites at the same amount of LG. However, the thermal conductivity values for dried WS-LG/TO composites were higher than for control WS because significant reduction is not possible due to structural changes caused by TO and LG’s ability to become soaked into internal pore structures, filling all cavities and cracks in WS and reducing the porosity of composites. Similar observations were made by Chalapud et al. [21] for TO coated particleboards from rise husk and soy protein, who stated that TO covers the external surface and can be located inside the particleboard samples. In Figure 2d, partially LG/TO coated WS pores can be depicted at 50 wt.% LG and 10 wt.% TO, while fully coated and filled pores of WS composites can be seen at 100 wt.% LG and 10 wt.% TO (Figure 2e). Interestingly, TO forms blister-like derivatives in the LG layer, thus additionally creating a polymer barrier due to conjugated double bonds in its structure [22,23].

Additionally, EG was incorporated in conjunction with LG for future flammability studies, and its impact on the thermal conductivity of dried WS composites was evaluated in Figure 1c. A total of 5 wt.% EG as well as 10 wt.% EG significantly increase the thermal conductivity independently of the amount of LG. The highest increment can be seen for 100 wt.% LG and 10 wt.% EG WS composites, and it is 6.3% greater compared to control WS. EG particles are the graphite compound with a high thermal conductivity, and due to their large size, which was reported by Liu et al. [24] to be 227 µm, EG simply lies on the surface of the WS particle at 50 wt.% LG (Figure 2f), and as shown, 50 wt.% was not sufficient to effectively cover the surface of the WS particle; some of the EG fell off or were only partially connected to WS. Although 100 wt.% LG made a solid layer on the surface of WS with a tight insert of EG (Figure 2g).

Moreover, chemical changes in the control WS and modified WS composites are shown in Figure 2j by means of FTIR spectra. The wide band observed between 3600 cm^−1^ and 3000 cm^−1^ is characteristic for stretching vibration of –OH due to inter- and intra-molecular hydrogen bonds [25] in control WS and modified WS composites. The transmittance band ranging from 2890 to 2900 cm^−1^ is caused by the stretching of –CH in the aliphatic and aromatic groups [26]. For WS-100LG/10TO and WS-100LG/10TO/10EG, these –CH stretching peaks at 2920 cm^−1^ and 2850 cm^−1^ were stronger than for other compositions due to additional fatty acids in TO. Furthermore, the C=O peak at around 1750 cm^−1^ is much stronger for coatings with TO oil, which can also be attributed to the existence of fatty acids. The peaks between 1590 cm^−1^ and 1570 cm^−1^ show C=C, which is characteristic of lignin in control WS and modified WS composites [26]. The peaks in control WS and modified WS composites represent the aliphatic deformation of –CH between 1315 cm^−1^ and 1370 cm^−1^ and come from hemicellulose or cellulose, while the –CO stretching peak at around 1034 cm^–1^ is for C–O–C in hemicellulose and cellulose. Compared to control WS, these peaks are stronger for all modified WS composites due to Si-O asymmetric stretching vibrations, which come from SiO_3_ groups present in the LG [27]. WS composites with LG in coating compositions also have additional transmittance peaks at around 1634 cm^−1^ [28] and 823 cm^−1^ [29], which are attributed to H_2_O trapped in Si-O-Si structures and Si-O, respectively.

The behavior of the thermal conductivity of dried WS-LG composites in Figure 1 can be described by regression equations further presented in Table 2. Standard deviations of respective descriptive equations are rather low, and determination coefficients are close to 1.

**Figure 3:**
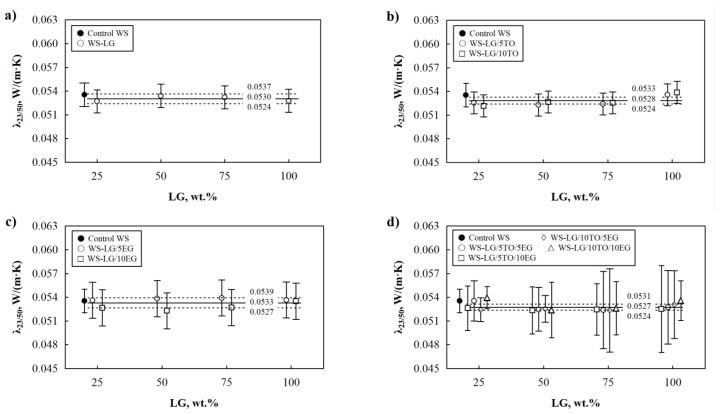
Thermal conductivity of WS-LG, WS-LG/TO, WS-LG/EG, and WS-LG/TO/EG composites at 23 °C and 50% relative air humidity: (**a**) WS-LG composites; (**b**) WS-LG/TO composites; (**c**) WS-LG/EG composites and (**d**) WS-LG/TO/EG composites.

As it was previously stated by Mussa and Salih [30] and Božiková et al. [31], wood and wood-based materials absorb moisture from the air under humid conditions, thus changing the thermal insulation performance of the resulting building materials. Figure 3a depicts the changes in the thermal conductivity values of control WS and WS-LG composites at 50% relative air humidity.

Control WS shows an increase in the parameter, while incorporating LG slightly reduces thermal conductivity values. Such changes can be explained by the fact that LG forms a thin layer on the surface of WS, thus blocking the hygroscopic sites in WS from contact with moisture. As Figure 2b shows, WS particles at 50 wt.% LG are not sufficiently coated, allowing the moisture to be absorbed and leading to an increased thermal conductivity value at 23 °C/50% of ~0.0534 W/(m·K). In comparison, WS-LG composite at 100 wt.% has an even and smooth surface with few air bubbles formed during drying, which interfere with the LG layer but slightly reduce thermal conductivity to ~0.0528 W/(m·K).

**Figure 4:**
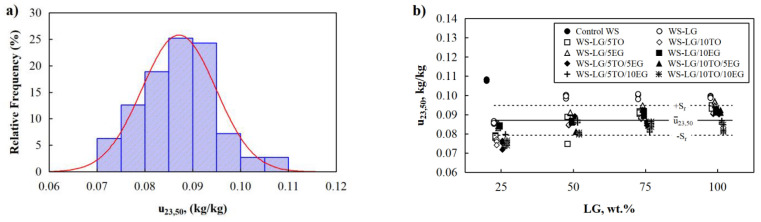
Experimental values of moisture content in WS-LG, WS-LG/TO, WS-LG/EG, and WS-LG/TO/EG: (**a**) histogram of normal distribution fitting; (**b**) average values of the moisture content.

Further, Figure 3b shows the impact of the LG and TO systems on the WS composites at 50% air humidity. The addition of 25–75 wt.% LG and 5–10 wt.% TO leads to an insignificantly lower thermal conductivity of WS-LG/TO composites compared to control WS, while 100 wt.% LG and 5–10 wt.% TO increase the parameter to the point that it becomes similar to the control WS. Overall, WS-LG/TO composites in humid conditions have unchanged thermal conductivity; the slight deviations in the results are insignificant. Similar trends can be seen in addition to 5–10 wt.% (Figure 3c) and for the LG/TO/EG system. According to the results obtained from the stationary zone of the weight gain of the samples, a histogram of relative frequencies was made (Figure 4a). The external view of the histogram allows us to hypothesize that a normal distribution can characterize the statistical distribution of the obtained research results. It was determined that the probability of the first type of error in weight gain is equal to 0.612.

This hypothesis about the correspondence of the results to the normal distribution with a confidence level of *p* = 0.95 was confirmed using the Pearson test. Therefore, there is no reason to reject the hypothesis about the normality of the analyzed data. According to the experimental test data, WS with LG and TO/EG have an average weight gain of 0.087 kg/kg and a mean square deviation of 0.00773 kg/kg (Figure 4b).

Based on the weight gain results for control WS, WS-LG, WS-LG/TO, WS-LG/EG, and WS-LG/TO/EG composites, it can be concluded that thermal conductivity at 23 °C/50% for all compositions is almost unaltered due to the hygroscopic nature of control WS and the increased hydrophobicity of LG, LG/TO, LG/EG, and LG/TO/EG coated WS composites. In other words, as control WS is more prone to moisture, its thermal conductivity increases, as Kol [32] and Troppová et al. [33] revealed that the thermal conductivity of pine wood significantly increases under different moisture contents. The moisture content in wood or wood particles increases water molecules within the wood or wood particles matrix. Therefore, humid wood’s thermal conductivity is higher than dried wood’s. Furthermore, the molecular spaces in the solid part of the wood or wood particles increase, thus giving greater molecular mobility and energy transportation. Further, coated WS composites are less susceptible to moisture due to LG, LG/TO, LG/EG, and LG/TO/EG coatings, and the increase in their thermal conductivity is not that rapid, resulting in insignificant fluctuations in thermal conductivity between control WS and coated WS composites.

### 3.2. Moisture Properties of Control WS and Modified WS Composites

**Figure 5:**
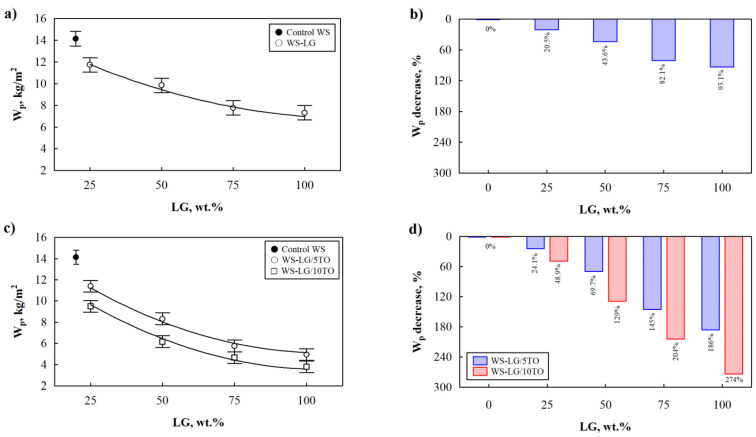

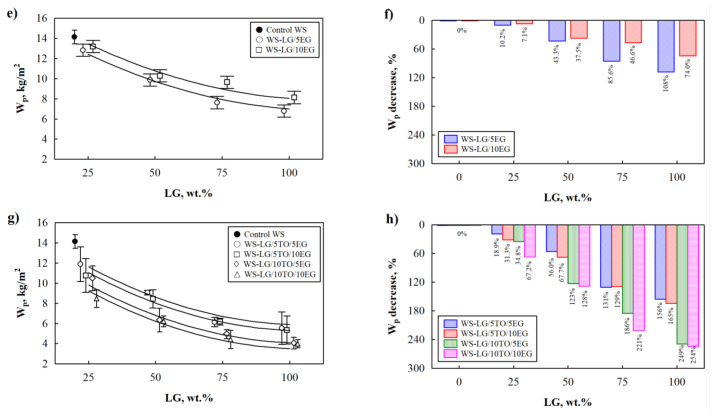
Water absorption of WS-LG, WS-LG/TO, WS-LG/EG, and WS-LG/TO/EG composites: (**a**,**c**,**e**,**g**) short-term water absorption by partial immersion; (**b**,**d**,**f**,**h**) percentage change of short-term water absorption.

As control WS and WS-LG composites mainly consist of polar WS particles, it is important to evaluate the moisture properties of such materials. Figure 5 presents the results of short-term water absorption and the percentage decrease in short-term water absorption of control WS, WS-LG, WS-LG/TO, WS-LG/EG, and WS-LG/TO/EG composites. It can be seen from Figure 5a that the control WS has the greatest water absorption value, i.e., 14.1 kg/m^2^. Adding LG onto the surface of WS particles significantly improved the parameter, and the reduction, as indicated in Figure 5b, is by 20.5% at 25 wt.% LG and 93.1% at 100 wt.% compared to control WS, showing that LG acts as a hydrophobic coating. Although the results show great improvement, at lower amounts of LG, the water molecules are still mainly bound via free hydroxyl groups [34,35] of WS particles and diffuse to the interface existing between WS particles and the LG due to insufficient coating, for instance, shown at 50 wt.% LG in Figure 2b.

The incorporation of TO (Figure 5c) resulted in even lower water absorption values of WS-LG/TO composites, indicating a synergy effect between two constituents, i.e., LG and TO. Compared to control WS, these two coatings reduced water absorption by a maximum of 186% and 274% at 100LG/5TO and 100LG/10TO, respectively (Figure 5d). Such improvements are comparable and even greater compared to the study by Humar and Lesar [36] of TO coating for spruce and beech woods, which resulted in a 100% improvement in water uptake at 100 wt.% TO. Moreover, compared to WS-LG composites at 100 wt.% LG and WS-LG/TO composites at 100 wt.% LG and 10 wt.% TO, water absorption was reduced by 55%. These improvements are related to the fact that TO is a drying oil. When oxygen molecules link to adjacent carbon-hydrogen bonds, hydroperoxide formation begins and causes linkage between bonds and fatty acid chains, resulting in polymerization and durable as well as hard films. The studies of different seed oils also confirmed the hydrophobic nature of hardened TO oil by Tang et al. [37] and Janesch et al. [8].

Additionally, the impact of EG incorporation on the water absorption of WS-LG composites is evaluated and presented in Figure 5e. The improvement in parameter shown before was reduced with an addition of even 5 wt.% EG. Compared to control WS, WS-25LG/5EG and WS-25LG/10EG composites have only slightly reduced water absorption values, i.e., by 10.2% and 7.1% at 5 wt.% EG and 10 wt.% EG, respectively. Compared with WS-100LG (Figure 5a) and WS-100LG/10EG (Figure 5e), the characteristic increased by 11%, indicating that water molecules may penetrate through the interface between EG particles and LG coating, thus gaining access to the surface of WS particles. The obtained results greatly agree with the results presented by Thirumal et al. [38] and Wrześniewska-Tosik et al. [39] who found out that water absorption increases at 10 wt.% EG in polymer matrix due to interconnection of cells and tearing as well as collapsing the cells in the polymeric cellular structure. Moreover, Figure 5g shows the impact of the LG/TO/EG system on the water absorption of WS composites, while Figure 5h shows the percentage decrease in water absorption of WS-LG/TO/EG composites. Depending on the amount of LG, TO, or EG, the water absorption of composites is reduced. The highest decrease can be observed for WS composites with 100 wt.% LG, 10 wt.% TO, and 10 wt.% EG.

At this composition, the parameter was reduced by a maximum of 254% compared to the control WS. Although WS-LG composites with added TO (Figure 5d) have a reduction in water absorption of 274% and WS-LG composites with incorporated EG (Figure 5f) only 74%, the composition WS-LG/TO/EG had a greater improvement in the parameter than it was expected indicating the synergy effect between TO and EG. It can be assumed that the interface between LG and EG was filled with TO, thus inhibiting the penetration of water molecules through the hydrophobic layers of LG and TO. The efficacy of various plant-based oils was also confirmed by a few research groups [40], which confirmed the hydrophobic behavior of the various hardened semi-drying and drying oil films, especially linseed and tung. The effective and smoother TO layer was obtained due to selected hardening conditions (23 °C temperature and 50% relative air humidity) because authors Huang et al. [41] reported a wrinkled surface of the TO film due to forced hardening conditions, i.e., higher temperature or UV light.

As Figure 5 shows, there are tendencies in which water absorption changes with the addition of LG, LG-TO, LG-EG, and LG/TO/EG. Therefore, such tendencies were described in the regression equations presented in Table 3. Standard deviations of respective descriptive equations are rather low, and determination coefficients are close to 1.

**Figure 6:**
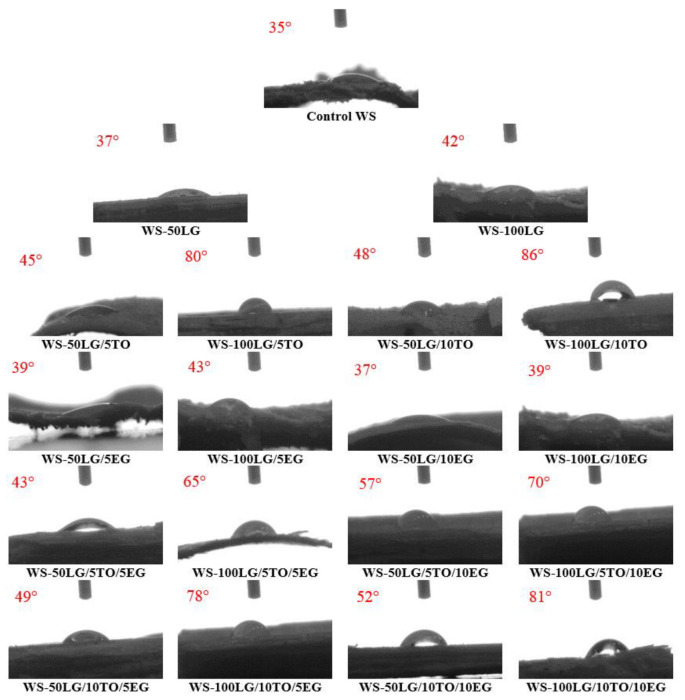
Water contact angle results of control WS and modified WS composites.

It is known [42] that the walls of the wood cells are composed of cellulose, etc., and that they have hydroxyl groups on their branch chains that tend to combine with water, thus creating hydrogen bonds that result in hydrophilicity. Therefore, the water resistance was also evaluated by the wettability test, expressed by contact angle measurements (Figure 6). For comparison, the compositions with 50 wt.% LG and 100 wt.% LG were chosen. Considering that water absorption (Figure 5) was significantly reduced for WS-LG composites, the water contact angle results are not so promising. For instance, 50 wt.% LG increased the contact angle by 2°, while 100 wt.% LG increased it by 7°. As He et al. [43] stated, due to triglycerides, which are hydrocarbon-rich, semi-drying and drying seed oil provide a coating that increases the hydrophobicity of wood or wood composite surfaces. The authors showed that control wood had a contact angle of 0° with a fully spread water drop on the surface, while TO-coated wood was characterized by a contact angle equal to 107°. It is not a surprise because the surface roughness of the control WS is assumed to be higher compared to the smooth coating of TO. Therefore, as the surface roughness increases, the contact angle of the WS surface gradually decreases [44]. Observations made by the current research showed that efficacy increases only at 100 wt.% LG and 5–10 wt.% TO, i.e., the parameter increased from 80° to 86° as indicated in Figure 6 due to the ability of LG coating to ultimately minimize the exposure of hydrophilic hydroxyl groups, thus increasing the hydrophobicity of WS-LG composites [45].

However, the incorporation of EG had an insignificant improvement of a few degrees independent of the amount of LG or EG used. As previously mentioned, the contact zone or insufficient interface between LG and EG on WS could be the cause of the almost unchanged surface wettability compared to control WS. Water contact angle measurements made by Hammi [46] revealed that the incorporation of 11 wt.% metallic filler in phosphate glass-based composites decreases the wettability of the surface by a maximum of 30%. The results are comparable with the current study, as EG increases contact angle results by a maximum of 23% at 100 wt.% LG and 5 wt.% EG.

Further, the addition of TO and EG improved the contact angle of WS-LG composites. The higher contact angles result from the different polarities of the water and surfaces. Water has a high polar part of its surface tension, as described in the Rzeczkowski et al. study [47], which indicates higher contact angles with non-polar surfaces such as LG, LG/TO, or LG/TO/EG on WS. As a result, a contact angle of 81° was achieved for WS-100LG/10TO/10EG composites, and so the obtained coating system is not fully hydrophobic.

**Figure 7:**
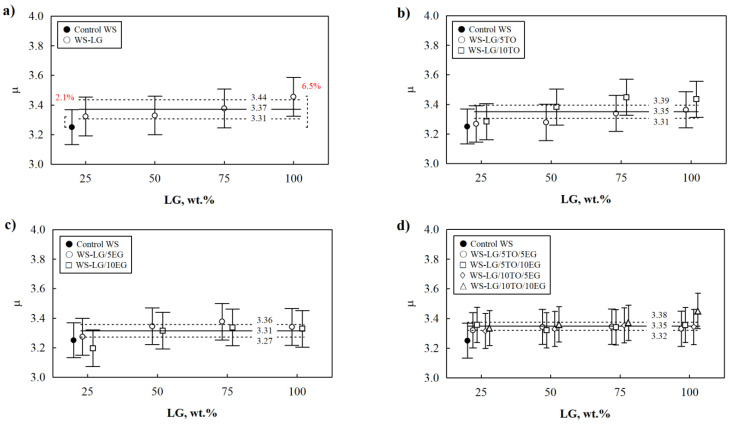
Water vapor permeability results: (**a**) WS-LG; (**b**) WS-LG/TO; (**c**) WS-LG/EG, and (**d**) WS-LG/TO/EG.

Water vapor permeability is one of the most important characteristics of thermally insulating materials used in building envelopes. There is insufficient data on the water permeability parameters of novel composites, so it is essential to determine the impact of LG, LG/TO, LG/EG, and LG/TO/EG coatings on the resulting values of the water vapor diffusion factor (Figure 7). With these results, this work may be the starting point for expanding water vapor permeability studies of various composites for the building industry, which are still scarce [48].

In all WS composites with different coatings, the water vapor diffusion factor is higher than the control WS. Even though the increase in the parameter is still observed, especially for the LG and LG/TO coatings, the difference between the obtained results is insignificant, which leads to the conclusion that even at the highest amounts of LG and LG/TO (Figure 7a,b), WS composites are still water vapor permeable. Further, the results presented in Figure 7a show that 100 wt.% LG increases the water vapor diffusion factor by 6.5%, while adding 10 wt.% EG at the same amount of LG–by only 2.5% (Figure 7c). These results indicate that the LG coating contributes significantly to increased permeability, and EG acts as a barrier for vapor penetration. Similar observations were made by Ilsouk et al. [49] and García et al. [50], who incorporated various fillers to increase the vapor sorption barrier effect in polymeric systems. The arrangement of particles in the coating could cause this behavior of water vapor permeability. The scattered filler particles result in water vapor following an aggravated path through the coating that surrounds the filler, in this case, EG particles.

## 4. Conclusions

The current study researched uncoated WS particles and LG, LG/TO, LG/EG, and LG/TO/EG coated WS composites as potential loose-fill thermal insulating materials for building envelopes. Considering the performance characteristics, optimal results were achieved with 100% WS, 100% LG, 10% TO, and 10% EG three-component coatings. The resulting coating system determined WS composites with thermal conductivity in the dry state with an average value of 0.048 W/(m·K), thermal conductivity under normal laboratory conditions of 0.054 W/(m·K), short-term water absorption of 4 kg/m^2^, contact angle of 81° and unchanged water vapor permeability characteristics compared to control WS samples. Additionally, regression equations were concluded describing the thermal conductivity and short-term water absorption behavior of the resulting WS composites depending on the amount of LG, TO, and EG. The synergy between components in three-component systems was achieved because 100% LG formed the smooth surface on WS particles, closing the access for water molecules to penetrate the hydrophilic sites of WS, 10% TO filled the gaps and cracks formed during the hardening of LG coating, and 10% EG could possibly contribute to reducing the flammability parameters of WS composites. Even though the results of the study were very promising, the agglomeration of coated WS particles occurred. Therefore, further investigations on how to avoid agglomeration after coatings are hardened should be considered. Although WS composites contained EG particles, further testing of flammability and ignitability should be taken into consideration. Therefore, future studies of the research group mentioned in this article will be dedicated to the evaluation of WS composites with regard to fire resistance.

## Figures and Tables

**Table 1:** Composition of WS-based loose-fill thermal insulation.

Sample	Component, % by Weight of WS			
	WS	LG	TO	EG
Control WS	100	0	0	0
LG solution				
WS-25LG	100	25	0	0
WS-50LG	100	50	0	0
WS-75LG	100	75	0	0
WS-100LG	100	100	0	0
LG/TO solution				
WS-25LG/5TO	100	25	5	0
WS-25LG/10TO	100	25	10	0
WS-50LG/5TO	100	50	5	0
WS-50LG/10TO	100	50	10	0
WS-75LG/5TO	100	75	5	0
WS-75LG/10TO	100	75	10	0
WS-100LG/5TO	100	100	5	0
WS-100LG/10TO	100	100	10	0
LG/EG solution				
WS-25LG/5EG	100	25	0	5
WS-25LG/10EG	100	25	0	10
WS-50LG/5EG	100	50	0	5
WS-50LG/10EG	100	50	0	10
WS-75LG/5EG	100	75	0	5
WS-75LG/10EG	100	75	0	10
WS-100LG/5EG	100	100	0	5
WS-100LG/10EG	100	100	0	10
LG/TO/EG solution				
WS-25LG/5TO/5EG	100	25	5	5
WS-25LG/5TO/10EG	100	25	5	10
WS-25LG/10TO/5EG	100	25	10	5
WS-25LG/10TO/10EG	100	25	10	10
WS-50LG/5TO/5EG	100	50	5	5
WS-50LG/5TO/10EG	100	50	5	10
WS-50LG/10TO/5EG	100	50	10	5
WS-50LG/10TO/10EG	100	50	10	10
WS-75LG/5TO/5EG	100	75	5	5
WS-75LG/5TO/10EG	100	75	5	10
WS-75LG/10TO/5EG	100	75	10	5
WS-75LG/10TO/10EG	100	75	10	10
WS-100LG/5TO/5EG	100	100	5	5
WS-100LG/5TO/10EG	100	100	5	10
WS-100LG/10TO/5EG	100	100	10	5
WS-100LG/10TO/10EG	100	100	10	10

**Table 2:** Results of statistical processing of thermal conductivity of dried modified WS composites.

Constant Coefficients in Equations					Standard Deviation S_r_	Determination Coeff. R^2^
b_0_	b_1_	b_2_	b_3_	b_4_		
WS-LG: $\lambda_{dry\to WS-LG}=b_{0}+b_{1}\cdot m_{LG}+b_{2}\cdot m_{LG}^{2}$						
0.04640	−2.125·10^−5^	2.99·10^−7^			0.000125	0.954
WS-LG/TO: $\lambda_{dry\to WS-LG/TO}=b_{0}+b_{1}\cdot m_{LG}+b_{2}\cdot m_{TO}+b_{3}\cdot m_{LG}^{2}$						
0.04699	−1.654·10^−5^	−9.542·10^−5^	2.210·10^−7^		0.000153	0.897
WS-LG/EG: $\lambda_{dry\to WS-LG/EG}=b_{0}+b_{1}\cdot m_{LG}+b_{2}\cdot m_{EG}+b_{3}\cdot m_{LG}^{2}$						
0.04554	−3.779·10^−5^	1.640·10^−4^	4.800·10^−7^		0.000193	0.954
WS-LG/TO/EG: $\lambda_{dry\to WS-LG/TO/EG}=b_{0}+b_{1}\cdot m_{LG}+b_{2}\cdot m_{TO}+b_{3}\cdot m_{EG}+b_{4}\cdot m_{LG}^{2}$						
0.04549	−9.702·10^−6^	6.058·10^−5^	1.063·10^−4^	2.117·10^−7^	0.000220	0.882

NOTE: The significance of the variables in the equations was determined according to the Student’s t-test at the 0.05 level of significance. Designations of variables in equations: m_LG_ is the amount of LG, wt.%; m_TO_ is the amount of TO, wt.%; and m_EG_ is the amount of EG, wt.%.

**Table 3:** Results of statistical processing of short-term water absorption of modified WS composites.

Constant Coefficients in Equations					S_r_	R^2^
**b_0_**	**b_1_**	**b_2_**	**b_3_**	b_4_		
WS-LG: $W_{p\to WS-LG}=b_{0}+b_{1}\cdot m_{LG}+b_{2}\cdot m_{LG}^{2}$						
14.807	−0.1336	5.787·10^−4^			0.480	0.947
WS-LG/TO: $W_{p\to WS-LG/TO}=b_{0}+b_{1}\cdot m_{LG}+b_{2}\cdot m_{TO}+b_{3}\cdot m_{LG}^{2}$						
17.208	−0.1986	−0.3170	9.400·10^−4^		0.472	0.971
WS-LG/EG: $W_{p\to WS-LG/EG}=b_{0}+b_{1}\cdot m_{LG}+b_{2}\cdot m_{EG}+b_{3}\cdot m_{LG}^{2}$						
14.966	−0.1604	0.2073	7.040·10^−4^		0.603	0.938
WS-LG/TO/EG: $W_{p\to WS-LG/TO/EG}=b_{0}+b_{1}\cdot m_{LG}+b_{2}\cdot m_{TO}+b_{3}\cdot m_{EG}+b_{4}\cdot m_{LG}^{2}$						
18.133	−0.1866	−0.3617	−0.1165	8.813·10^−4^	0.550	0.955

NOTE: The significance of the variables in the equations was determined according to the Student’s t-test at the 0.05 level of significance. Designations of variables in equations: m_LG_ is the amount of LG, wt.%; m_TO_ is the amount of TO, wt.%; and m_EG_ is the amount of EG, wt.%.

## Data Availability

Not applicable.
